# Global and reflective rumination are related to suicide attempts among patients experiencing major depressive episodes

**DOI:** 10.1186/s12888-021-03119-z

**Published:** 2021-02-26

**Authors:** Hao Tang, Tingting Xiong, Jiabo Shi, Yu Chen, Xiaoxue Liu, Siqi Zhang, Huan Wang, Qing Lu, Zhijian Yao

**Affiliations:** 1 Department of Psychiatry, Affiliated Nanjing Brain Hospital, Nanjing Medical University, No. 264 Guangzhou Road, Nanjing, 210029 Jiangsu China; 2 School of Biological Sciences & Medical Engineering, Southeast University, No. 2 Sipailou Road, Nanjing, 210096 Jiangsu China; 3 Child Development and Learning Science, Key Laboratory of Ministry of Education, Nanjing, China; 4 Nanjing Brain Hospital, Medical School, Nanjing University, Nanjing, China

**Keywords:** Major depressive disorder, Bipolar disorder, Rumination, Suicide attempt, Risk factor

## Abstract

**Background:**

Recent attention has focused on the role of rumination in suicidality, with evidence indicating that rumination may be positively related to suicidal ideation. There remains disagreement on the nature of the relationship between rumination and suicide attempts, especially in major affective disorders. This study was designed to identify whether rumination is a risk factor for attempted suicide.

**Methods:**

A total of 309 patients with major depressive episodes were recruited for this study, including 170 patients with major depression and 139 patients with bipolar disorder. All participants were categorized into two groups based on a series of clinical assessments: suicide attempters (*n* = 87) and non-suicide attempters (*n* = 222). Rumination was evaluated with the Ruminative Responses Scale. A binary logistic regression analysis was carried out to evaluate the relationship between rumination and suicide attempts.

**Results:**

Both global ruminative levels and the two subtypes of rumination, brooding and reflection, were significantly higher in the suicide attempters than the non-suicide attempters. After controlling for age, current depression and anxiety symptoms, and episode frequency, it was found that global rumination and reflection (but not brooding) were positively associated with suicide attempts.

**Conclusion:**

These results suggest that rumination may be a risk factor for suicide attempts and highlight the maladaptive nature of reflection in patients with major depressive episodes.

## Introduction

Suicide is a tremendous challenge for global public health, with close to 800,000 lives lost to suicide every year [1]. Between half and two-thirds of all completed suicides are committed by people suffering from mood disorders [2]. Patients with major affective disorders, especially those diagnosed with major depressive disorder (MDD) and bipolar disorder (BD), are at very high risk of death by suicide, with a lifetime prevalence of attempted suicide of around 31 and 33.9%, respectively [3]. As a proposed lethality measure, the ratio of suicide attempts is also comparable in MDD and BD, at 10.2 and 11.1, respectively [4]. Accordingly, identification of the risk factors for suicide attempts in major affective disorders may improve suicidal risk recognition and contribute to targeted interventions. Several clinical and psychological indicators, such as depression, hopelessness, and impulsivity, are good predictors of suicidal ideation [4, 5]. However, the critical vulnerabilities that predict the risk of suicide attempts have not yet been fully elucidated.

In recent years, rumination has been suggested as a potential cognitive factor related to suicide. According to Nolen-Hoeksema’s Response Styles Theory [6], rumination is described as a stable trait response pattern in which individuals passively and repetitively focus on the reasons, consequences, and symptoms of their distress rather than engage in proactive problem-solving. Rumination is related to malicious excessive self-referential processes and is generally regarded as a maladaptive cognitive style due to its repetitive and passive thinking properties. It can exacerbate psychopathology and interfere with dynamic approach behavior [7]. Rumination has been linked with prolonged negative mood and aggravated depressive symptoms in non-clinical individuals [8, 9], along with the course of the disease and episode number in both MDD and BD patients [10, 11].

Treynor and colleagues [12] described two principal components of rumination: brooding and reflection. Brooding is characterized by dwelling on the outcomes of negative emotions, while reflection pertains to pondering the reasons for a depressed mood. It has been suggested that brooding may be a maladaptive process, while reflection may be an adaptive strategy because it may facilitate problem-solving in the long run; therefore, studies should assess global rumination and both aspects of rumination separately. Rumination has been shown to be a good predictor of the presence and duration of suicidal ideation [13–15]. In contrast, there is limited and inconclusive evidence regarding the association between rumination (both global rumination and its two subsets) and suicide attempts, especially in relation to depressive episodes [16].

In a non-clinical cross-sectional survey of 1696 college students, brooding was significantly related to suicide risk and attempts [17]. Valderrama et al. [18] found an indirect relationship between brooding and suicidal behavior, independent of reflection; however, Surrence and colleagues failed to observe differences in brooding and reflection between suicide attempters and non-attempters in a study of 96 college undergraduates. In contrast, reflection appears to be positively associated with suicidal thoughts in individuals with a history of suicide attempts [19]. Krajniak et al. [20] followed up 143 college students for 2–3 years, 32 of whom had a history of suicide attempts. Those who attempted suicide showed higher levels of suicidal ideation at follow-up, and this association was mediated by global rumination.

When it comes to clinical studies, global rumination was found to be a core symptom of “suicidal syndrome” in a study of 2383 schizophrenia patients and 1920 mood disorder patients; however, these findings were not specific to major mood disorders [21]. Psychiatric inpatients reporting a history of suicide attempts had higher brooding levels than non-suicidal individuals, even after controlling for patients’ depressive symptoms [22]. A longitudinal study of 286 individuals seeking psychiatric emergency services found no significant cross-sectional or prospective correlations between global rumination and suicide attempts. In contrast, brooding, rather than reflection, was related to a lifetime history of suicide attempts; however, brooding could not predict future suicide attempts [23]. Similarly, in a cross-sectional study of 83 suicide attempters admitted to an Accident and Emergency department, Cameron et al. [24] found ruminative brooding was directly correlated with both suicide attempts and ideation accompanied by current low mood.

The literature reviewed above suggests that suicide attempts may be related to global rumination and brooding, rather than reflection. This assertion is supported by a recent meta-analysis showing moderate associations between suicide attempts and both global rumination and brooding (Hedge’s g = 0.26 and 0.47, respectively), but no association between suicide attempts and reflection (Hedge’s g = 0.09) [16]. Nonetheless, these findings should be interpreted with caution and require further refinement as few evaluated clinical samples. Furthermore, there is remarkable heterogeneity among the samples from the limited available clinical studies, which may affect the findings’ reliability and limit their generalizability. In the published clinical studies to date, most participant samples comprised patients with mood disorders, together with patients with schizophrenia, schizoaffective disorder, substance abuse, or other confounding factors [21, 22, 25]. There is currently no available data specific to major depressive episodes.

This study aimed to investigate whether rumination is a risk factor for suicide attempts in major affective disorder. Rumination is prevalent in both depression and bipolar disorder, meaning the patients’ negative self-concept/schemas might be similar during the depressive episodes which are characteristic of each disorder [11, 26, 27]. To improve sample homogeneity, we only included patients with major depressive episodes to eliminate the influence of other mental diseases or mixed symptoms. We put forward two hypotheses: 1) suicide attempters would display a higher degree of rumination, including global rumination and both subcategories of rumination, than non-attempters; and 2) global rumination and brooding would be the prominent risk factors for suicide attempts among patients suffering from major depressive episodes.

## Methods

### Study design

This study adopted a retrospective design. Data, including demographic data, clinical assessments, and rumination assessments, were collected from patients diagnosed with major affective disorders.

### Participants

The sample patients were recruited from those hospitalized at the Affiliated Brain Hospital of Nanjing Medical University between June 2019 and May 2020. Patients were enrolled who met the diagnostic criteria of the Statistical Manual of Mental Disorders, 4th Edition, Text Revision (DSM-IV-TR) [28] for major depressive disorder or bipolar disorder with current major depressive episode. The inclusion criteria were: (1) confirmed diagnosis according to the Chinese version of the Mini-International Neuropsychiatric Interview (MINI) [29]; (2) Han Chinese ethnicity aged 16–65 years with a junior high school education or above; (3) 17-item Hamilton Rating Scale for Depression (HRSD-17) score [30] > 7; (4) Young Mania Rating Scale (YMRS) [31] score < 5; (5) Brief Psychiatric Rating Scale (BPRS) score [32]<35; and (6) no structured psychotherapy within 6 months. The exclusion criteria included current mania/hypomania episode and mixed state of bipolar disorder, comorbidity with other mental disorders, psychotic symptoms, severe and unstable physical disease, and pregnancy or lactation.

Three hundred sixty-nine consecutively admitted patients were screened in the initial study. Fifty-five patients were excluded as they did not meet the inclusion criteria, including 10 with mixed episodes, seven with a comorbid personality disorder, 12 with psychotic symptoms, 12 with a comorbid anxiety disorder, three with comorbid obsessive-compulsive disorder, five with comorbid alcohol dependence, and six with only primary school education. In total, 314 participants were enrolled in the study, of which four were excluded as too much data were missing from their self-assessment scales. Thus, a total of 309 subjects were included in the statistical analyses. The local ethics committee approved this study, and all subjects gave written informed consent before participating.

### Measures

A suicide attempt is defined as self-harm behavior with an inferred or actual intention to die [33]. Each patient’s history of suicide attempts was evaluated as part of the clinical interview upon admission to the hospital. These data were obtained from the patient’s medical records and also covered in the MINI and HRSD assessments. Further, the patient’s responses to the Nurse’s Global Assessment of Suicide Risk (NGASR) [34] confirmed their suicide attempt history. The NGASR is a structured assessment to assess suicide risk; the Chinese version has demonstrated good reliability and validity [35]. The NGASR comprises 15 questions. The 7th item requires the doctor to ask “if there was a history of prior suicide attempt’; this item is scored 0 for “NO” and 3 for “YES.” Based on the above data, participants were divided into two groups: suicide attempters (SA) and non-suicide attempters (NSA).

The Ruminative Responses Scale (RRS) is the most commonly used measure of rumination. It contains a 22-item self-report inventory which is a revision of the Response Styles Questionnaire [36]. The Chinese version of the RRS, which has demonstrated reliability among Chinese college students and patients with major depression [37, 38], was employed in the present study. The RRS is composed of three dimensions: brooding, reflection, and depression-related rumination. Brooding and reflection are considered the main components of rumination, while depression-related rumination overlaps more with depressive symptoms and is often removed [12]. We used the brooding and reflection sub-scores (five items each) and the total score (the sum of the brooding and reflection scales, reflecting global rumination) as the candidate risk factors predicting suicide attempts in the subsequent analyses.

As mentioned above, a series of measurement methods evaluated participants’ clinical symptoms. The HRSD-17, Hamilton Anxiety Rating Scale (HAMA) [39], YMRS, and BPRS were used to assess each patient’s severity of depression, anxiety symptoms, manic symptoms, and psychotic symptoms, respectively. All assessments were completed within 3 days of the patient’s hospitalization.

### Statistical analyses

Data management and analyses were performed using SPSS 23.0. Two-sample t-tests, non-parametric tests, or chi-square analyses were utilized to compare the groups with respect to demographic data, clinical data, and the RRS and its subscale scores. Partial correlations (with Bonferroni correction) were performed to explore the relationships between rumination and each major clinical variable. Next, binary logistic regression was used to predict whether ruminative thinking was a risk factor for suicide attempts. To control for the effects of clinical factors as much as possible, demographic and clinical variables with a *P*<0.1 in the first step of the single factor analysis were selected as covariates for the regression analysis. The total and factor scores of the RRS were analyzed separately and the VIF value evaluated to test for multicollinearity. Further, the Hosmer-Lemeshow test was used to assess the goodness of fit of the logistic regression models.

## Results

### Demographic and clinical data

The full cohort included 87 suicide attempters and 222 non-attempters; 170 were diagnosed with MDD and 139 with BD. As shown in Table 1, the groups did not differ significantly by education or gender; however, the mean age of suicide attempters was significantly lower than non-attempters (U = 7335.500, *P* = 0.001). There were no significant differences between the two groups with respect to HRSD, HAMA, YMRS, BPRS, disease duration, episode frequency, family history, and MDD/BD diagnosis. The *P* values for HRSD (*t* = − 1.725, *P* = 0.086) and episode frequency (U = 8467.500, *P* = 0.082) were less than 0.1, so these variables were included as covariates, along with age, in the subsequent regression analyses. Given the close relationship between anxiety and suicide [40], we also included HAMA as an independent variable. Suicide attempters showed much higher NGASR scores than non-attempters; however, because of the intrinsic relationship between suicide risk and attempts, the NGASR scores were not included in the regression analysis.

**Table 1:** Characteristics of the study sample

Characteristic		SA (*n* = 87)	NSA (*n* = 222)	*χ*^*2*^ /*t*/*U*	*P*
Gender	Male/Female	30/57	92/130	1.267	0.260 (c)
Age (years)	Median (IQR)	23 (16)	32 (23)	7335.500	0.001** (u)
Education (years)	Mean (SD)	13.77 (3.54)	13.69 ± 3.49	2.677	0.102(t)
Course (months)	Median (IQR)	36.00 (72.00)	36.00 (76.00)	9514.500	0.840(u)
No. of episodes	Median (IQR)	2.00 (2.00)	2.00 (2.00)	8467.500	0.082(u)
Family history of mental disease	Yes/No	33/54	63/159	2.663	0.103(c)
Family history of suicide	Yes/No	7/80	10/212	1.508	0.267(c)
Diagnosis	MDD/BD	42/45	128/94	2.223	0.136(c)
HDRS	Mean (SD)	22.11 (4.48)	21.07 (5.54)	−1.725	0.086(t)
HAMA	Mean (SD)	15.28 (5.87)	14.68 (6.18)	−0.304	0.762(t)
YMRS	Mean (SD)	2.26 (1.40)	2.18 (1.34)	−0.514	0.608(t)
BPRS	Mean (SD)	24.17 (2.14)	24.09 (2.30)	−0.272	0.785(t)
NGASR	Mean (SD)	11.02 (2.57)	5.78 (3.04)	−14.234	0.000*** (t)
No. of suicide attempts	*n* (%)				
1		52 (59.8%)			
2		25 (28.7%)			
≥3		10 (11.5%)			
Suicide attempt immediately before admission	*n* (%)	27 (31.0%)			

*MDD* Major Depression Disorder, *BD* Bipolar Disorder, *SA* Suicidal attempters, *NSA* Non-suicide attempters, *HDRS* Hamilton Depression Rating Scale, *HAMA* Hamilton Anxiety Rating Scale, *YMRS* Young Mania Rating Scale, *NGASR* Nurse’s Global Assessment of Suicide Risk, *SD* Standard deviation, *IQR* Inter quartile range

Comparisons were conducted using *t*-tests (t), Mann-Whitney U tests (u), and Chi-squared tests (c)

*P < 0.05, ** *P* < 0.01, ***P < 0.001

### The relationships between rumination and major clinical variables

Cronbach’s α was first calculated to test the reliability of the RRS in the current sample. The internal consistency was found to be adequate for the global rumination score (α = 0.857) and both brooding (α = 0.782) and reflection (α = 0.734) scores. As can be seen from Table 2, the suicide attempters reported significantly higher levels of global rumination, brooding, and reflection compared to the non-attempters.

**Table 2:** Comparison of rumination between SA and NSA groups

		SAs	NSAs	*t*	*P*
Brooding	Mean (SD)	14.01 (2.99)	12.24 (3.27)	−4.374	0.000***
Reflection	Mean (SD)	12.54 (2.68)	10.58 (2.92)	−5.436	0.000***
GR	Mean (SD)	26.55 (5.10)	22.82 (5.76)	−5.287	0.000***

*SA* Suicidal attempters, *NSA* Non-suicide attempters, *GR* Global rumination, *SD* Standard deviation

*P < 0.05, ** *P* < 0.01, ***P < 0.001

Research suggests that rumination may be affected by age and sex [41, 42]; therefore, a partial correlation analysis controlling for sex and age was used to characterize the correlations between rumination and HRSD, HAMA, course of the disease, and episode frequency. There were positive relationships between global rumination and both HRSD (*r* = 0.250, *P* = 0.000) and episode frequency (*r* = 0.193, *P* = 0.001). Brooding was positively correlated with HRSD (*r* = 0.252, *P* = 0.000), while reflection was significantly related to HRSD (*r* = 0.207, *P* = 0.000) and episode frequency (*r* = 0.183, *P* = 0.001). The threshold for a significant correlation was set at *P* < 0.002, Bonferroni corrected (Table 3).

**Table 3:** Correlations between rumination and major clinical variables

Control Variables			GR	Brooding	Reflection	HDRS	HAMA	Episodes	Course
Gender & Age	GR	Correlation	–						
		Significance	–						
	Brooding	Correlation	0.931	–					
		Significance	0.000*	–					
	Reflection	Correlation	0.915	0.705	–				
		Significance	0.000*	0.000*	–				
	HDRS	Correlation	0.250	0.252	0.207	–			
		Significance	0.000*	0.000*	0.000*	–			
	HAMA	Correlation	0.144	0.160	0.102	0.489	–		
		Significance	0.012	0.005	0.074	0.000*	–		
	Episodes	Correlation	0.193	0.174	0.183	0.034	0.072	–	
		Significance	0.001*	0.002	0.001*	0.554	0.209	–	
	Course	Correlation	0.123	0.107	0.121	−0.025	−0.067	0.545	–
		Significance	0.031	0.061	0.034	0.664	0.240	0.000*	–

*HDRS* Hamilton Depression Rating Scale, *HAMA* Hamilton Anxiety Rating Scale, *GR* Global rumination

*Significant at *P*-value < 0.002 (Bonferroni corrected)

### Association between rumination and suicide attempts

**Fig. 1:**
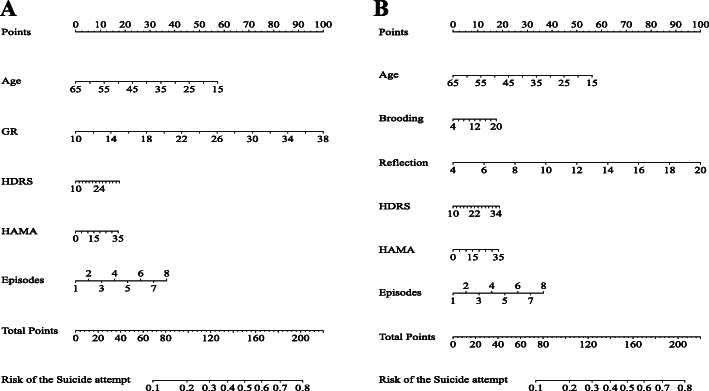
Nomogram to estimate the risk of suicide attempt. Figures A and B show the relationships between suicide attempts and both GR and its subcomponents, respectively. Each risk factor can be vertically correlated to the corresponding “Points.” “Total Points” sums up the “Points” for all risk factors to match the “Risk of the suicide attempt”. GR, Global Rumination

As mentioned above, HRSD, HAMA, episode frequency, and age were included as covariates in the binary logistic regression to explore the relationship between rumination and suicide attempts. Consistent with our hypothesis, global rumination was significantly associated with suicide attempts [OR (95% CI) = 1.101 (1.046, 1.159), *P* = 0.000]. In terms of the rumination subtypes, patients with higher levels of reflection were more likely to report a history of suicide attempts [OR (95%CI) = 1.184 (1.042, 1.345), *P* = 0.009]. In contrast, brooding was not a statistically significant predictor of suicide attempt history [OR (95% CI) = 1.030 (0.916, 1.158), *P* = 0.622]. Table 4 presents the logistic regression summary statistics. Based on these findings, a nomogram was configured (Fig. 1).

**Table 4:** Logistic regressions examining concurrent associations

–	B	S.E.	Wald	OR	95% CI	*P*	Nagelkerke R^2^	VIF	Hosmer-Lemeshow χ^2^
							0.156		13.085
GR	0.096	0.026	13.517	1.101	1.046–1.159	0.000***		1.194	
Age	−0.031	0.012	6.295	0.970	0.946–0.993	0.012*		1.156	
HDRS	0.018	0.030	0.383	1.019	0.961–1.079	0.536		1.388	
HAMA	0.013	0.026	0.260	1.013	0.963–1.066	0.610		1.382	
Episodes	0.142	0.092	2.355	1.152	0.962–1.380	0.125		1.045	
	B	S.E.	Wald	OR	95% CI	*P*	Nagelkerke R^2^	VIF	Hosmer-Lemeshow χ^2^
							0.162		7.986
Brooding	0.030	0.060	0.243	1.030	0.916–1.158	0.622		2.152	
Reflection	0.169	0.065	6.749	1.184	1.042–1.345	0.009**		2.144	
Age	−0.030	0.012	6.066	0.970	0.947–0.994	0.014*		1.157	
HDRS	0.020	0.030	0.431	1.020	0.962–1.081	0.512		1.388	
HAMA	0.014	0.026	0.300	1.014	0.964–1.067	0.584		1.386	
Episodes	0.141	0.092	2.341	1.152	0.961–1.381	0.126		1.045	

*GR* Global rumination, *HDRS* Hamilton Depression Rating Scale, *WALD* Wald statistic, *S.E.* Standard error, *VIF* Variance inflation factor, *OR* Odds ratio, *CI* Confidence interval

*P < 0.05, ** *P* < 0.01, ***P < 0.001

## Discussion

This study was designed to determine the relationship between rumination and suicide attempts in a sample of hospital inpatients with major depressive episodes. Consistent with our hypothesis, depressive suicide attempters exhibited significantly higher rumination (including its subtypes, brooding and reflection) than non-attempters. However, it was somewhat surprising that global rumination and reflection, not brooding, were associated with suicide attempts, even when controlling for the severity of depression and anxiety symptoms.

The RRS total score provides an overall measure of global depressive rumination. Consistent with our findings, Fazakas-Dehoog et al. [43] found that suicide attempters had higher RRS scores than non-attempters in a non-clinical sample. Likewise, Yaseen et al. [44] found that psychiatric patients with a history of suicide attempts showed higher levels of ruminative flooding and regard rumination as a vital factor of the “suicide trigger state.” Theoretically, there may be a persuasive pathological association between rumination and suicide attempts. Repetitive negative thinking may result in helplessness and temptation, which could generate suicidal thoughts; then, the transition from considering suicide to attempting suicide could be promoted by repeated exposure to suicidal thoughts and imagery [45]. In one study, at baseline, suicide attempters scored higher on global rumination than non-attempters and exhibited increased vulnerability to suicidality at a two-year follow-up [20]. Our results broaden the evidence that global rumination is linked with suicide attempts in clinical samples [16], with our study broadening those findings to patients with major depressive episodes.

The most striking result of this study is that reflection, rather than brooding, was associated with suicide attempts. Traditionally, it has been argued that reflection is associated with increased depression concurrently and decreased depressive symptoms longitudinally [12], meaning it may have a protective effect to some extent [46]. However, there are other possible interpretations of the role of reflection.

Several studies have found associations between reflection and suicidal ideation [47] or a history of both non-suicidal self-injury and suicide attempts [25], suggesting that reflection may at times be a negative coping strategy. Reflection involves a tendency to understand why one feels distressed; however, if repetitive attempts are fruitless, a person could fall into negative thinking cycles, resulting in impaired mood regulation and problem-solving [48]. From another perspective, reflection might be adaptive only if there are no negative cognitive biases [49]. Furthermore, in another study, reflection was positively associated with suicidal intent in suicide attempters with low problem-solving skills [50]. These studies present potential mechanisms underlying our finding of reflection as a risk factor for suicide attempts in the current sample of participants suffering from a major depressive episode. We speculate that reflection when suffering negative emotions might increase vulnerability to everything from suicidal thoughts to suicide attempts.

Another important finding was, that although the brooding and reflection scores of the suicide attempters were higher than the non-attempters, the regression analysis did not find brooding to be a significant risk factor for attempted suicide. This outcome is contrary to previous findings which have suggested that brooding is cognitively maladaptive and more strongly associated with a history of suicide attempts than reflection in psychiatric inpatients [17, 22]. The reason for this unexpected finding is not apparent but may have something to do with the critical contribution of brooding to clinical symptoms. These results are in accord with Surrence’s study [19] which found that brooding did not predict suicide attempts. These authors argued that the impact of brooding on suicide is primarily through an increase in depressive symptoms and hopelessness. Our research also found that brooding was only related to the severity of depression as measured by various clinical indicators; thus, brooding may contribute more to the depressive symptoms which somehow influence suicide. However, caution must be applied when interpreting these findings which should be replicated in mood disorders to develop a full picture and confidence in the generalizability of the findings.

The present study is one of the first attempts to examine the relationship between rumination and suicide attempts in patients with major affective disorders. These results may offer valuable insight into the vital role of ruminative thinking in attempted suicide in depression. Understanding the link between rumination and suicide will further highlight the importance of rumination assessment in clinical practice, regardless of MDD or BD diagnoses. The results indicate that for depressed patients with high levels of global rumination and reflection, clinicians must pay close attention to the determination of suicide risk during treatment. Specifically, interventions that focus on alleviating rumination, such as Rumination-focused Cognitive Behavioural Therapy [51], might be particularly beneficial for suicide prevention in patients with depression.

### Limitations

Several limitations of this research must be noted. Firstly, it is now well established there is high psychiatric comorbidity in both MDD and BD [52, 53]. Stricter inclusion criteria were adopted in this study to eliminate the influence of comorbidity, which resulted in a relatively small sample size. Another source of weakness was the cross-sectional design of this study, which might undermine the determination of causality. A longitudinal investigation is required to determine whether initial levels of rumination are reliable predictors of future suicide attempts in major affective disorder, especially given that some MDD patients may convert to BD and be challenged by manic/hypomanic episodes over time. Thirdly, we concentrated only on the significance of rumination and did not measure the associations between suicide attempts and other risk factors, such as hopelessness and impulsivity [5]. Additionally, structural and functional changes in specific brain regions may be involved in both rumination and suicide [54]. The predictive efficacy of rumination for suicide attempts may be significantly improved when combined with comprehensive clinical assessment and neuroimaging techniques, such as rumination-related neurophysiological or functional imaging.

## Conclusion

Overall, the current study demonstrates a critical role of global rumination and reflection in suicide attempts. Notwithstanding the relatively limited sample size, this work offers valuable insight into the relationship between rumination and suicide attempts in major depressive episodes. Suicide attempters exhibited higher rumination scores (both global scores and scores for each subtype); however, only global rumination and reflection were substantially correlated with suicide attempts after controlling for depression and anxiety levels. The current data add to the growing body of evidence indicating that rumination confers a risk for suicide attempts and expands our understanding of maladaptive attributes of reflection in major affective disorders. Future studies using a broader range of rumination assessments could shed more light on the role of rumination in predicting suicidal behavior in clinical practice.

## Data Availability

The datasets generated during the current study are not publicly available due to the subjects’ privacy but are available from the corresponding author on reasonable request.

## Abbreviations

BD: Bipolar Disorder
BPRS: Brief Psychiatric Rating Scale
DSM-IV-TR: Statistical Manual of Mental Disorders, 4th Edition, Text Revision
HAMA: Hamilton Anxiety Rating Scale
MDD: Major Depressive Disorder
MINI: Mini-International Neuropsychiatric Interview
NGASR: Nurse’s Global Assessment of Suicide Risk
NSA: Non-suicide attempters
RRS: Ruminative Responses Scale
SA: Suicide attempters
YMRS: Young Mania Rating Scale
